# Evaluating antimalarial efficacy by tracking glycolysis in *Plasmodium falciparum* using NMR spectroscopy

**DOI:** 10.1038/s41598-018-36197-3

**Published:** 2018-12-24

**Authors:** Rupali Shivapurkar, Tejashri Hingamire, Akshay S. Kulkarni, P. R. Rajamohanan, D. Srinivasa Reddy, Dhanasekaran Shanmugam

**Affiliations:** 1 Biochemical Sciences Division, CSIR-National Chemical Laboratory, Pune, India; 2 Academy of Scientific and Innovative Research (AcSIR), New Delhi, India; 3 Organic Chemistry Division, CSIR-National Chemical Laboratory, Pune, India; 4 Central NMR facility, CSIR-National Chemical Laboratory, Pune, India

## Abstract

Glucose is an essential nutrient for *Plasmodium falciparum* and robust glycolytic activity is indicative of viable parasites. Using NMR spectroscopy, we show that *P. falciparum* infected erythrocytes consume ~20 times more glucose, and trophozoites metabolize ~6 times more glucose than ring stage parasites. The glycolytic activity, and hence parasite viability, can be measured within a period of 2 h to 5 h, using this method. This facilitates antimalarial bioactivity screening on ring and trophozoite stage parasites, exclusively. We demonstrate this using potent and mechanistically distinct antimalarial compounds such as chloroquine, atovaquone, cladosporin, DDD107498 and artemisinin. Our findings indicate that ring stage parasites are inherently more tolerant to antimalarial inhibitors, a feature which may facilitate emergence of drug resistance. Thus, there is a need to discover novel antimalarial compounds, which are potent and fast acting against ring stage parasites. The NMR method reported here can facilitate the identification of such molecules.

## Introduction

Malaria is a devastating infectious disease, and the human malaria parasite *P. falciparum* is responsible for ~500,000 annual deaths worldwide^1^. Clinical symptoms associated with human malaria manifest during blood stage schizogony, which is initiated when the parasite invades a red blood cell (RBC) and undergoes a 48 h developmental cycle, culminating in RBC lysis and release of daughter parasites called merozoites that are capable of invading a new RBC. Following RBC invasion, the parasite matures into a series of morphologically distinct forms called ring, trophozoite and schizont stages, before the formation of daughter merozoites and exiting the red cell^2^. The rapid growth and proliferation of the blood stage parasite is supported by robust metabolism of glucose as a source of carbon and energy *via* glycolysis^3^. Since glycolysis is an essential metabolic pathway for malaria parasite survival, glycolytic activity is therefore a good indicator of parasite viability^4,5^.

Prior work has shown that glycolytic activity of live *P. falciparum* infected RBC (iRBC) can be monitored in real time by nuclear magnetic resonance (NMR) spectroscopy, by measuring lactate accumulation over time^6^. We reasoned that this method can be used to evaluate the effect of anti-malarial compounds on distinct intra-erythrocytic stages of the malaria parasite, specifically ring *vs* trophozoite stages. Trophozoites are metabolically very active as they prepare for cell division by schizogony, which makes them more vulnerable to various anti-malarial drugs and inhibitors. Compared to trophozoites, ring stage parasites are metabolically less active, and are likely less affected by antimalarial compounds^7^. Recent findings indicate that dormancy of ring stage parasites, can contribute to the development of artemisinin resistance^8,9^. Thus, it is critical to understand the differential response of distinct intra-erythrocytic stages of the parasite to antimalarial interventions.

**Figure 1:**
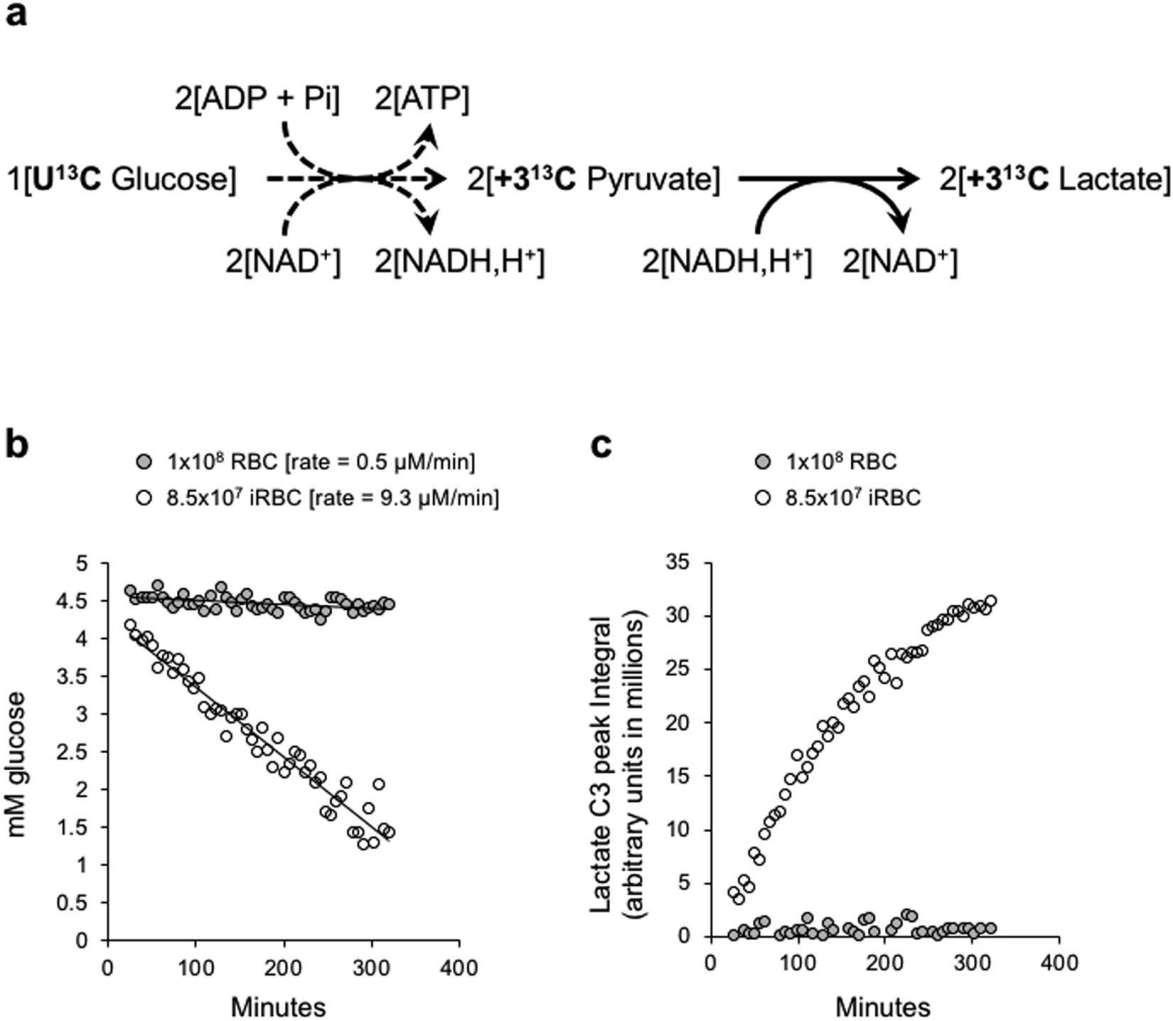
Real time measurement of glycolytic activity from live RBC and iRBC by ^13^C NMR. (**a**) Schematic representation of stoichiometric conversion of U^13^C-glucose to U^13^C-lactate. U^13^C-glucose utilization (**b**) and U^13^C-lactate production (**c**) by RBCs (grey filled circles) and iRBCs (open circles), respectively, were measured. In case of glucose absolute quantification in mM concentration was determined using glucose standard (Supplementary Fig. S1d), while for lactate, the raw integral value for the carbon 3 (C3) peak was plotted for each time point. The rate at which glucose is metabolized by RBCs and iRBCs is derived from the slope of the linear portion of the respective fits. Data shown in **b** and **c** were obtained from the same experiment.

In this study, we have generated a high-resolution profile of glycolysis for the complete 48 h developmental cycle of the intra-erythrocytic forms of *P. falciparum*. This was done by monitoring the conversion of U^13^C-glucose into U^13^C-lactate (Fig. 1a) by live *P. falciparum* infected human erythrocytes, using ^13^C NMR spectroscopy. The differences in glycolytic rate between ring and trophozoite stage parasite can be clearly observed from this NMR experiment. The short time periods required to measure glycolytic rate of ring and trophozoite stage parasites allowed us to monitor the effect of potent antimalarial compounds on these stages of the parasite. This is important, since standard antimalarial screening methods^10–13^ do not explicitly look at intraerythrocytic developmental stage specific parasite killing, as these are mostly end point assays designed to determine inhibition of parasite growth over a two or three-day period. Moreover, it is necessary to distinguish between inhibitors, which merely arrest parasite growth as opposed to killing it (i.e., static *vs* cidal). By using NMR to measure parasite glycolytic activity in the presence of various mechanistically distinct inhibitor compounds, we show that ring stage parasites are inherently tolerant to potent antimalarial compounds.

## Results

### Tracking glycolytic activity in live *P. falciparum* infected RBCs by ^13^C-NMR

For real time monitoring of glycolysis in *P. falciparum* infected RBCs (iRBCs), we developed a protocol for directly tracking glycolytic activity in iRBCs by NMR. A detailed description of this protocol is given in the Materials and Methods section. From the NMR spectral profile we could clearly visualize the ^13^C peaks corresponding to all 6 carbons of glucose (substrate for glycolysis), and all 3 carbons of lactate (end product of glycolysis) at the expected chemical shift values (Supplementary Fig. S1a to S1c). The stacked time series spectral profiles clearly indicate the progressive decrease in the signals corresponding to glucose while those for lactate show progressive increase over the course of NMR experiment (Supplementary Fig. S1a). Based on the integral value of the NMR spectra obtained for a dilution series of pure U^13^C-glucose standard samples (1 mM to 5 mM) (Supplementary Fig. S1d), we were able to estimate the rate at which RBCs (~10^8^ cells) and iRBCs (~8.5 × 10^7^ cells) metabolize glucose to lactate (Fig. 1b). This experiment was carried out with trophozoite stage iRBCs enriched from *P. falciparum* cultures using a 60% percoll cushion, in order to avoid the background signal from uninfected RBCs, which constitute ~90% of total cells. The corresponding NMR data obtained from RBCs and iRBCs for C6 of glucose was plotted as a linear fit, and the slope of the fit was used as a measure for the rate of U^13^C-glucose consumption. The consumption of glucose by iRBCs (9.3 µM/min) was ~20 times higher than that of RBCs (0.5 µM/min) (Fig. 1b). The higher glycolytic flux in iRBCs, when compared to RBCs, was also confirmed from the kinetics of lactate formation (Fig. 1c).

### Dynamics of glycolytic activity during intra-erythrocytic developmental cycle of *P. falciparum*

**Figure 2:**
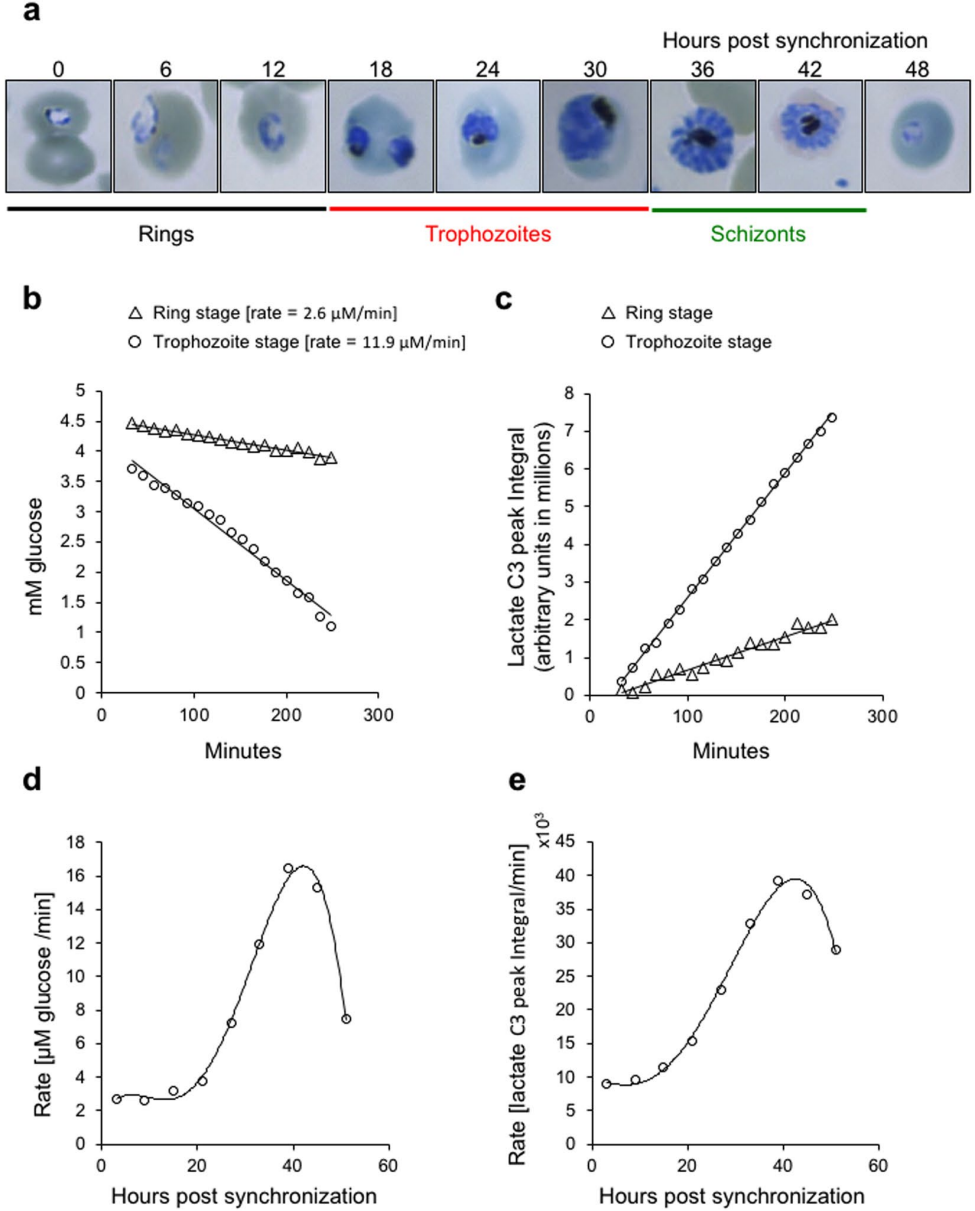
Temporal profiling of glycolytic activity during the 48 h intraerythrocytic development of *P. falciparum*. (**a**) Giemsa stained smears showing the different developmental stages of *P. falciparum* at 6 h intervals following sorbitol synchronization of the parasites. U^13^C-glucose metabolism (**b**) and U^13^C-lactate production (**c**) by ring stage (culture sampled from 0.5 hps to 6 hps) and late trophozoite stage (culture sampled from 30 hps to 36 hps) parasites respectively. Data was plotted as in Fig. 1. Glycolytic profile of iRBCs during the complete 48 h intraerythrocytic developmental cycle is plotted as either the rate of glucose depletion (**d**) or rate of lactate formation (**e**), respectively.

During erythrocytic schizogony, *P. falciparum* undergoes a ~48 h developmental cycle in which the parasite transits through morphologically and physiologically distinct stages known as ring, trophozoite and schizont. The lytic cycle ends when schizonts mature to merozoites and egress out of iRBCs. Merozoites initiate a new cycle by immediately invading a healthy RBC (Fig. 2a). Distinct molecular events, supported by stage specific gene and protein expression, are known to occur throughout this developmental cycle^14^. The trophozoite stage parasite is metabolically more active than other intra-erythrocytic stages^15^. We therefore studied the dynamics of glycolytic activity at 6 h intervals, for the entire intraerythrocytic developmental cycle of *P. falciparum* by NMR. For this, we started with a population of highly synchronized iRBC population containing >90% ring stage parasites. ^13^C-NMR measurements were obtained from cultures sampled at 0.5, 6, 12, 18, 24, 30, 36, 42 and 48 h post synchronization (hps). ~3 × 10^8^ total cells (at 10–15% parasitemia) from synchronized *P. falciparum* cultures sampled at the indicated time points were re-suspended in 500 µl media containing 5 mM U^13^C-glucose, and were subject to NMR profiling over a period of 6 h. It should be noted that, in this and all subsequent experiments described, the complete culture i.e., mixture of RBC and iRBC, was used for NMR analysis. The integrals from resonances corresponding to ^13^C6- of glucose and ^13^C3- of lactate for each time point were plotted to obtain linear fits, and the slope of the fit was used to derive the rate of U^13^C-glucose utilization (Fig. 2b) or U^13^C-lactate production (Fig. 2c). The rates determined for glucose utilization and lactate production from all time points were plotted to generate a glycolytic profile for the complete 48 h intraerythrocytic developmental cycle of the parasite. This revealed that trophozoite stage parasites exhibit higher glycolytic activity in comparison to ring stage parasites (Fig. 2d,e).

### Comparing the viability of chloroquine treated ring and trophozoite stage *P. falciparum* by monitoring their glycolytic activity

The ability of ring stage parasites to survive drug exposure and facilitate the development of drug resistance has been documented^8,9^. Thus, it is likely that ring stage *P. falciparum* is more tolerant to antimalarial drugs in comparison to trophozoite stage parasites. Therefore, it is important to have a method for monitoring the independent response of ring and trophozoite stage parasites to antimalarial compounds. As parasite viability is intimately linked to an active glycolytic flux, decreased glycolytic activity is a good indicator of decreased parasite viability. We reasoned that live cell NMR measurements of glycolytic activity can be useful for tracking the viability of malaria parasites in real-time. Since this NMR method allows glycolytic activity (and hence viability) profiling within a short duration (i.e., 2–6 h), it can facilitate viability studies on ring and trophozoite stage parasites exclusively, following their exposure to antimalarial drugs.

**Figure 3:**
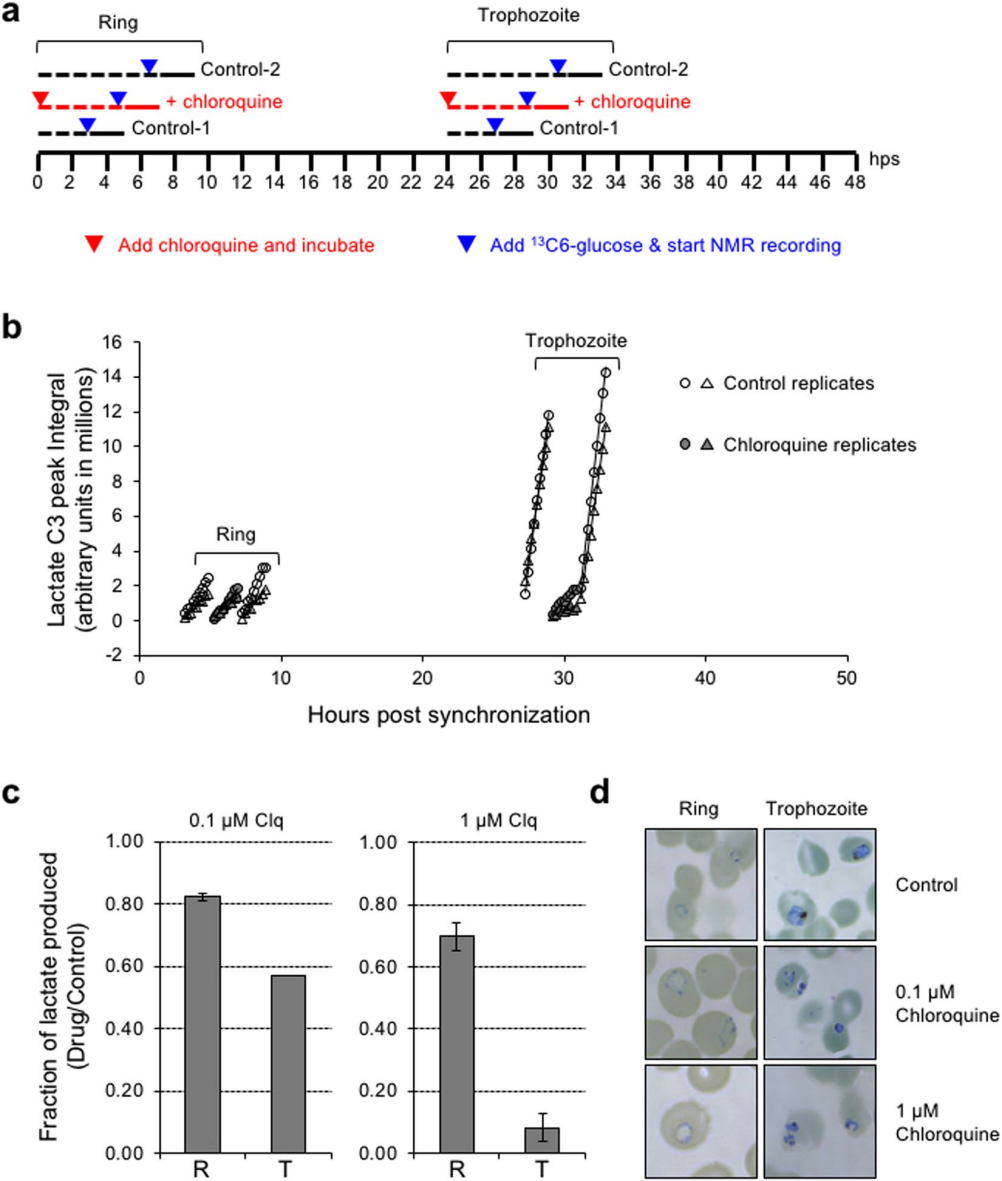
Evaluating the effect of chloroquine on glycolytic activity of ring and trophozoite stage *P. falciparum*. (**a**) Schematic representation of the experimental set-up. The scale represents the 48 h period following sorbitol synchronization of the iRBCs. The three lines shown above this scale indicate control (black) and drug (red) treated samples. In each line, the dashed part indicates the incubation time before addition of U^13^C-glucose (blue inverted triangles) and the bold part indicates the 2 h period during which the NMR measurements were taken. The red inverted triangles indicate the time at which the drug was added. Parasites were treated with drug for a total of 7 h (5 h before and 2 h during NMR measurements). hps, hours post synchronization. (**b)** Plot of the lactate C3 peak integral values for ring and trophozoite stage parasites measured for 2 h by NMR. Data from two replicate experiments are overlaid; individual replicate data is provided in Supplementary Fig. S4. (**c)** The plots depict the fold-change in glycolytic rate in ring (R) and trophozoite (T) stage parasites, in the presence of 0.1 µM and 1 µM chloroquine, respectively. All the experiments were performed in duplicates and the error bars represent standard deviation for drug *vs* control. (**d**) Giemsa stained smears of cultures recovered after NMR measurement showing the morphology of iRBCs from control and chloroquine (0.1 µM and 1 µM) treated ring and trophozoite stage parasites, respectively.

We tested the feasibility of conducting such stage specific drug sensitivity assays using the potent antimalarial drug chloroquine. We designed the following protocol for this purpose, mindful of the fact that it is not feasible for us to subject drug treated and equivalent (i.e., developmental time matched) control cultures to NMR measurements in parallel due to the availability of a single 700 MHz NMR spectrometer in our institutional facility. A schematic overview of the experiment is shown in Fig. 3a. Sorbitol synchronized iRBC cultures containing >90% ring stage parasites, were divided into three equal parts (1 ml each at ~2% hematocrit and ~10–15% parasitemia). Two of these parts served as control cultures, while one was subject to treatment with the drug of choice (in this case chloroquine). Drug treatment was started immediately after synchronization, i.e., 0 hps. At 3 hps, one of the control cultures was taken for ^13^C-NMR profiling, for a period of 2 h. Following this, the drug treated culture and the second control culture were subject to NMR profiling sequentially from 5 hps to 7 hps, and 7 hps to 9 hps, respectively. 5 mM U^13^C-glucose was added to each of the samples 15 min prior to start of NMR acquisition. A similar protocol was followed for trophozoite stage parasites, which were sampled starting at ~24 hps, using the same batch of synchronized culture from which ring stage parasites were sampled (Fig. 3a). To ascertain that keeping parasites in the NMR machine for 2 h does not affect the viability of the parasite, we recovered the control parasites (which were not treated with inhibitors) after NMR measurements and again cultured them under optimal conditions for up to 72 hps, and found no significant loss in viability of the parasites (Supplementary Fig. S2). While monitoring glycolytic activity of iRBCs for 2 h periods, we found that our analysis was more reliable and consistent when monitoring U^13^C-lactate, rather than U^13^C-glucose. This is likely due to the fact that there is no preexisting U^13^C-lactate, and only what is produced by iRBCs during the experiment is measured. On the other hand, U^13^C-glucose is present at 5 mM concentration at the beginning of NMR measurements, and a small change in its levels due to consumption by iRBCs over a 2 h period, especially in case of ring stage parasites, is not reliably detected. We therefore used U^13^C3-lactate data to track glycolysis and parasite viability in all inhibitor treatment studies. The rate of lactate production is derived from slope values of the linear fits of the raw NMR peak integral data.

Chloroquine is known to act by interfering with and inhibiting heme polymerization in the food vacuole of the malaria parasite^16^. Thus, the target of this drug is a physiological process that is active in the trophozoite stage of the parasite. In the chloroquine sensitive 3D7 parasite strain used in this work, we determined the *EC*_50_ of the drug to be ~13 nM (Supplementary Fig. S3a). To ensure that parasites are exposed to a lethal dose of chloroquine in our NMR experiment, we used the drug at 100 nM and 1 µM concentration, which are both >EC_90_ levels. It was clearly evident from the intensity of NMR peak corresponding to C3 of lactate that glycolytic activity in trophozoite stage parasite is dramatically reduced in the presence of chloroquine, while there is only a marginal effect on lactate production in ring stage parasites (Fig. 3b and Supplementary Fig. S4a to S4d). The fold change in rate of lactate production between chloroquine treated and untreated parasites is shown in Fig. 3c. In trophozoite stage parasites, lactate production was decreased by ~40% and ~92%, following 5 h treatment with 100 nM and 1 µM chloroquine, respectively. Ring stage parasites, however, showed only ~20% and ~30% reduction in lactate production, for similar treatments with chloroquine. Figure 3d shows Giemsa stained smears of representative iRBCs from corresponding cultures. These results clearly validate the fact that ring and trophozoite stage parasites have differential response to chloroquine, and this can influence parasite clearance or residual persistence in infected host following drug treatment.

### Differential viability of ring and trophozoite stage *P. falciparum* exposed to potent anti-malarial compounds

**Figure 4:**
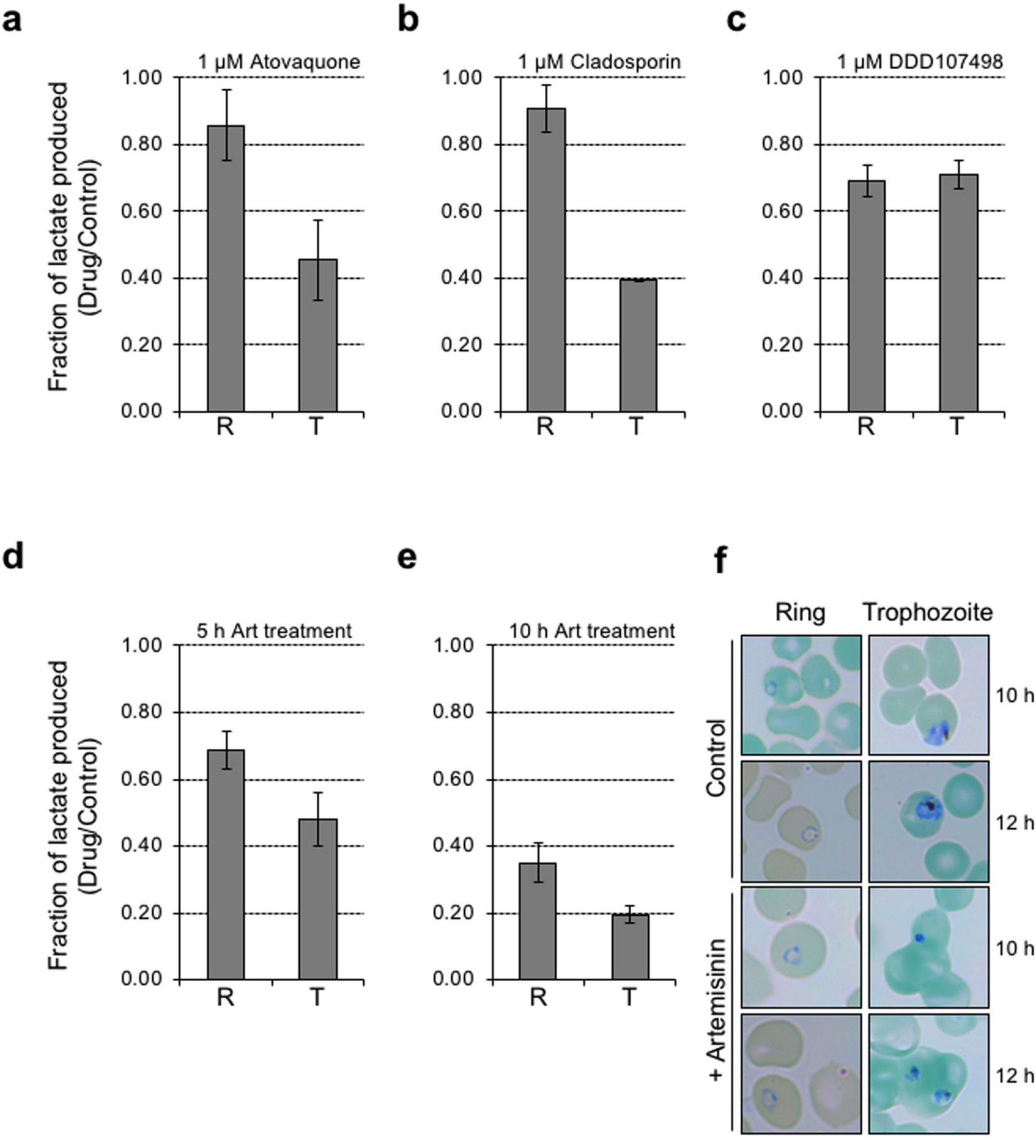
Evaluating the effect of various potent antimalarial compounds on glycolytic activity of ring and trophozoite stage *P. falciparum*. The plots depict the fold-change in glycolytic rate as measured for ring (R) and trophozoite (T) stage parasites treated with 1 µM each of atovaquone (**a**), cladosporin (**b**), and DDD107498 (**c**). In case of artemisinin, treatment was done for two different time periods of 5 h (**d**) and 10 h (**e**). The raw data for lactate C3 peak integral values, for ring and trophozoite stage parasites, from 2 h NMR measurements, from replicate experiments, is shown in Supplementary Fig. S4. All the experiments were performed in duplicates and the error bars represent standard deviation for drug vs control. (**f**) Giemsa stained smears showing the morphology of control and artemisinin treated (10 h) ring and trophozoite stage parasites, before (10 h) and after (12 h) NMR measurements.

The ability to clearly distinguish the effect of chloroquine on glycolytic activity and viability of ring and trophozoite stage parasites by NMR, encouraged us to investigate the effect of other potent antimalarial compounds by this method. For this, we choose two compounds already in use as antimalarial drugs, artemisinin^17^ and atovaquone^18^, and two experimental compounds with potent antimalarial activity, DDD107498^19^ and Cladosporin^20^. The anti-parasitic activity of these molecules has been studied in detail, and all are known to effect parasite clearance rapidly with *EC*_50_ values ranging from 1 nM to 100 nM^21^. The estimated *EC*_50_ values for the parasite strain used in this study is given in Supplementary Fig. S3b to S3d. Despite their potency, clinical resistance has been reported for artemisinin and atovaquone^22^. Therefore, we were interested in finding out whether there is any difference in the efficacy of these molecules against ring and trophozoite stage parasites. We essentially tested all four compounds in our NMR assay, using an identical protocol as used in the chloroquine experiment. The results from these assays are shown in Fig. 4 and Supplementary Fig. S4e to S4n. For all four inhibitors, the morphology of ring and trophozoite stage parasites, at 5 h (before NMR analysis) and at 7 h (after NMR analysis) is shown in Fig. S4.

Atovaquone, cladosporin and DDD107498 are mechanistically distinct in their antimalarial activity. Atovaquone inhibits CYTb protein of mitochondrial respiratory complex III^23^, DDD107498, a substituted quinolone compound with potent multistage antimalarial activity is known to act *via* inhibition of *P. falciparum* EF1α^19^, and cladosporin is a fungal metabolite and potent inhibitor of parasite lysyl tRNA synthetase^20^. We performed the NMR experiments to find out whether any of these molecules can kill ring stage parasites rapidly and in comparable manner to trophozoite stage parasites. In case of atovaquone and cladosporin, we once again observed more inhibition of glycolytic activity in trophozoite stage parasites (~52% and ~61% respectively) while only a minimum inhibition was observed in case of ring stage parasites (~15% and ~9% respectively) (Fig. 4a,b). Interestingly, we observed comparable inhibition of glycolytic activity in ring and trophozoite stage parasites (~32% and ~29% respectively) with the novel antimalarial compound DDD107498 (Fig. 4c).

Previous studies have shown that ring stage *P. falciparum* can escape artemisinin treatment and facilitate the emergence of drug resistance parasites^9^. We found that following 5 h artemisinin treatment, ring stage parasites showed ~30% reduction in lactate production, while trophozoite parasites showed ~50% reduction in lactate production (Fig. 4d). We then tested artemisinin effect on parasite glycolytic activity after a prolonged treatment time of 10 h. Following extended incubation with the drug, we found increased inhibition of glycolytic activity in both ring stage (~65% reduction in lactate production), and trophozoite stage (~80% reduction in lactate production) parasites (Fig. 4e). The morphology of ring and trophozoite stage parasites after 10 h and 12 h of artemisinin treatment (i.e., before and after NMR analysis) is shown in Fig. 4f. As can be seen from microscopic examination, the morphology of ring stage parasites appears to be normal even after 12 h artemisinin treatment, even though glycolytic activity appears to be reduced by >60%.

## Discussion

Owing to the emergence and spread of resistance to frontline antimalarial drugs, there is a need to discover novel inhibitors as leads for future drugs for malaria. In particular, antimalarial compounds that are effective against all intraerythrocytic developmental forms of the parasite need to be identified. Following invasion of a naive RBC, the merozoite stage of the malaria parasite initiates a 48 h asexual replication cycle, during which it sequentially develops into ring, trophozoite and schizont stages, before finally turning into merozoites and egressing out of the red cell to complete the cycle. A subset of the blood stage parasites turn into gametocytes, *via* a multi-step maturation process, which facilitates parasite transmission *via* the mosquito vector^24^.

Malaria parasites appear to have the innate ability to develop resistance to various inhibitory molecules, and this has been observed in the field and in the laboratory^25^. There is now substantial evidence suggesting that ring stage dormancy, i.e., temporary arrest of growth for many days or even weeks, helps malaria parasites recover from exposure to lethal doses of artemisinin based antimalarial drugs^8^. This can possibly be avoided by including a compound that can rapidly kill and eliminate ring stage parasites, as a partner drug in artemisinin based combination therapy (ACT). However, finding such a compound is not trivial, since the parasite transits through the ring stage development within the first 15 h following red cell invasion and most antimalarial screening methods are designed as end point assays to monitor parasite death after 48 h to 72 h, i.e., typically into the second invasion cycle, following the addition of drug. Dedicated assays to monitor the transitioning of ring stage parasites to trophozoite stage parasites, by microscopy and flow cytometry are available. However, while these assays are useful for monitoring ring to trophozoite transition, they do not provide information regarding the viability of the parasite with respect to each stage. In this study, we have developed a ^13^C-NMR spectroscopy method for tracking the stage specific effect of various antimalarial compounds by quantifying parasite glycolytic activity in real-time using live *P. falciparum* infected human red cells.

Glycolysis is an essential metabolic function in *P. falciparum*^4,5^ and the parasites have an intrinsically high rate of glycolytic flux^26^. When U^13^C-glucose is used as the major carbon source in parasite culture, its oxidation *via* glycolysis results in the formation of U^13^C-lactate^27^. In this study, we have developed an NMR protocol for real-time kinetic measurement of glycolytic flux in live *P. falciparum* cultures by monitoring U^13^C-glucose consumption and U^13^C-lactate production by the parasite. Using this method, we have successfully profiled glycolytic activity at 6 h intervals throughout the 48 h intra-erythrocytic asexual development of the parasite. In agreement with previous reports, we found that ring stage parasites exhibit minimal glycolytic activity, which progressively increased with time, as the parasites develop from ring to trophozoite, and then to schizont stages, before again decreasing to ring stage activity levels coinciding with the beginning of a new invasion cycle. The sensitivity of the NMR method allowed us to estimate glycolytic activity within a period of 2 h to 6 h, and thus made it possible to monitor parasite viability in intra-erythrocytic developmental stage specific manner, in the presence of antimalarial compounds.

In case of chloroquine, which targets hemoglobin degradation in the food vacuole, a prominent activity in the trophozoite stage, we observed a distinct trophozoite specific inhibition of parasite glycolytic activity, which was more pronounced at 1 µM drug (>90% inhibition; Fig. 3). Surprisingly, we observed only a 30% reduction in ring stage glycolytic activity, even at the high concentration of 1 µM drug. It should be noted that, upon incubation of ring stage parasites in 0.1 µM drug for 24 h or beyond, we noticed complete clearance of the parasite (Supplementary Fig. S6), suggesting that as the ring stage parasites mature into trophozoites, they are killed by the drug. We then tested the effect of four other potent antimalarial compounds, atovaquone^18^, cladosporin^20^, DDD107498^19^ and artemisinin^17^, each at 1 µM concentration. Interestingly, both atovaquone (a potent inhibitor of oxidative phosphorylation) and cladosporin (a potent inhibitor of lysyl tRNA synthetase) had very little effect on ring stage parasite glycolytic activity. DDD107498 (also an inhibitor of protein synthesis) was only moderately effective in inhibiting trophozoite stage glycolytic activity, but interestingly, ring stage parasites were also equally affected. With artemisinin, even though we observed comparatively better inhibition of glycolytic activity in trophozoite stage parasite, when the drug incubation time was increased to 10 h, we noticed a marked loss of glycolytic activity in ring and trophozoite stage parasites, suggesting that ring stage parasites are affected by longer duration of exposure to the drug. This may also reflect the fact that ring stage parasites are maturing to trophozoites, and then succumbing to drug effects.

In summary, using real-time measurements of glycolytic activity by NMR in *P. falciparum* infected human red cells, we demonstrate that it is possible to track drug mediated killing of blood stage parasites within a few hours. By virtue of the short time periods needed for these experiments, it is possible to study the drug effect on specific intraerythrocytic developmental stages of the parasite, such as ring and trophozoite. Our studies reveal that ring stage parasites have an inherent ability to better tolerate antimalarial drugs in comparison to trophozoites, and this can probably facilitate ring stage latency and emergence of drug resistance. The NMR assay method described here can facilitate the identification of novel antimalarial compounds that are capable of rapidly killing ring stage *P. falciparum*. In addition, this method can possibly be adapted for antimalarial efficacy studies on the other dominant human malaria parasite *P. vivax*, which cannot be maintained in long-term culture in the laboratory.

## Materials and Methods

### Preparation of stock and working solutions of antimalarial compounds

10 mM stock solutions of the atovaquone, artemisinin, cladosporin, and 5.18 mM stock solution of DDD107498 were prepared in cell culture grade DMSO (Sigma-Aldrich, US) and stored in −20 °C until use. 10 mM stock solution of the chloroquine was prepared in sterile cell culture grade water (Life Technologies, USA). 100 µM working stocks of these drugs were prepared and used in parasite killing assays. 5 µl from a 10 mM stock of atovaquone, artemisinin, cladosporin, and 9.7 µl from a 5.18 mM stock of DDD107498, was mixed with 495 µl and 490.3 µl DMSO to make 100 µM working stock solutions of respective compounds. 5 µl from 10 mM stock of chloroquine was added to 495 µl of sterile water to make 100 µM working stock solution of chloroquine.

### *P. falciparum* culture methods

All experiments involving the malaria parasite *P. falciparum* were reviewed and approved by the institutional biosafety committee of CSIR-National Chemical Laboratory, Pune. 3D7 strain of *P. falciparum* was used in all experiments. Routine culturing of intraerythrocytic stages of the parasite was carried out as reported^28^. Human RBCs required for culturing the *P. falciparum* was obtained from Poona Serological Trust Blood Bank, Pune, following appropriate guidelines. Blood was collected at the blood bank from anonymous adult voluntary donors after obtaining informed consent. Blood was collected in collection bags contained EDTA to prevent clotting. The protocols for collecting human blood and using it for culturing *P. falciparum* were approved by the institutional biosafety and human ethics committees of CSIR-National Chemical Laboratory, Pune. All reagents used for *P. falciparum* culture were purchased from Life Technologies, USA. The composition of the complete RPMI (cRPMI) medium used for culturing the parasite is as follows: 1x RPMI-1640 liquid medium was supplemented with HEPES (25 mM), gentamicin sulphate (10 mg/lit), glutamine (0.75 mM) and sodium bicarbonate (18 mM). Before use, the culture media was supplemented with Albumax II (0.5% w/v) and hypoxanthine (100 mg/lit). Parasite cultures were maintained in a humidified 37 °C incubator with 5% CO_2_. O +ve erythrocytes were used and the hematocrit of the culture was maintained at 2%.

The parasitemia was maintained around 5% routinely, and parasites were synchronized by 5% sorbitol treatment for 10 minutes, followed by washing with cRPMI. Sorbitol treatment will kill trophozoite and schizont stage parasites while sparing the ring stage parasites^28^. For some experiments, trophozoite and schizont stage parasites were isolated using a 60% percoll cushion^29^. Sorbitol treatment was carried out every alternate cycle to obtain a highly synchronous population of ring stage parasites, which were used in drug assays. Cells were counted using the Countess cell counter (Life Technologies, USA). Parasite growth was regularly monitored by examination of Giemsa stained thin blood smears. Progression through the different stages during intraerythrocytic development was monitored by microscopy and validated by flow cytometry^13^. For the antimalarial compounds used in this study the *EC*_50_ values were determined as reported previously^30^.

### U^13^C-glucose labeling of *P. falciparum* infected and uninfected RBCs

To monitor the glycolytic flux in *P. falciparum* infected and uninfected RBCs, the cells were labeled with U^13^C-glucose (Cambridge Isotopes, USA). For ^13^C- labeling experiments, Dulbecco’s modified Eagle’s medium (DMEM) minus glucose was used as the base medium, which was supplemented with all additives similar to that for cRPMI. Following two cycles of treatment with 5% sorbitol, highly synchronized ring stage cultures were obtained, as ascertained by flow cytometry, and this was used for the NMR experiments. For initial standardization of the method, percoll purified trophozoite stage parasites were used for increased signal detection. However, since the glycolytic activity of iRBCs was found to be many fold higher than that of uninfected RBCs, in subsequent experiments the whole infected population (i.e., RBC and iRBCs) was used. The parasitemia of the culture was maintained between 10% to 15% and the hematocrit was ~2% in all experiments.

For profiling glycolytic activity during the complete intraerythrocytic developmental cycle, 1 ml culture aliquots were sampled 0.5 hours post synchronization (hps) and then at every 6 h time interval thereafter. For inhibitor assays, the synchronized culture was split into six 1 ml aliquots, of which three were sampled at ring stage, while the other three were sampled at trophozoite stage. The total number of cells taken for NMR measurements varied between 5 × 10^7^ to 5 × 10^8^ for different experiments. For ^13^C labeling experiments, the cells were harvested from 1 ml culture aliquots by centrifugation and re-suspended in 500 µl culture media containing 5 mM of U^13^C-glucose and transferred to a 5 mm Wilmad glass NMR tube, and placed into an NMR spectrometer to acquire NMR spectra for the required time period. A D_2_O (Euriso-top, France) capillary (Wilmad, USA) was used to lock the field frequency and Trimethylsilylpropanoic acid (TSP; Euriso-top, France) was used as the internal chemical shift reference. The series of carbon NMR spectra were acquired sequentially for the required time period. The instruments used were either Bruker Avance 500 MHz or the Bruker Avance III HD 700 MHz NMR spectrometers.

### Assay method for assessing antimalarial activity by monitoring parasite glycolytic activity

Sorbitol synchronized cultures containing >90% ring stage parasites, were divided into six aliquots of 1 ml each, and three each were used for ring and trophozoite assays respectively. Out of the three culture aliquots, two served a controls and one was treated with the antimalarial compound, either immediately after synchronization (ring stage assay) or at 24 hps (trophozoite stage assay). Glycolytic activity was monitored in control cultures at 3 hps and 7 hps (ring stage assay), and 27 hps and 31 hps (trophozoite stage assay) respectively. Glycolytic activity was monitored for antimalarial compound treated cultures at 5 hps (ring stage assay) and 29 hps (trophozoite stage assay), respectively. A schematic outline of this protocol is depicted in (Fig. 3a).

### Acquisition and processing of ^13^C NMR data

The Bruker Avance 500 MHz NMR spectrometer was equipped with a 5 mm Broad-Band observe probe, and the observe frequency used for carbon was 125 MHz. For 1D ^13^C NMR experiments, the zgpg30 pulse program was used with a spectral width of 248.46 ppm and acquisition time of 1.04 sec at 310°K. The values for excitation pulse and the delay between the pulses were 12.25 μs and 2 sec, respectively. 128 transients were acquired in 6 minutes with 65 K data points. We used the Topspin (Version 3.5) program on Bruker Avance III HD 700 MHz NMR spectrometer equipped with 5 mm Broad-Band inverse probe. The ^13^C NMR spectra were acquired with zgpg pulse program. The acquisition time of 0.78 sec, spectral width 236.65 ppm, an excitation pulse of 10 *μ*s (~70°), and a relaxation delay of 5 sec between consecutive pulses, were used. 128 transients were acquired in 12 minutes with 65 K data points. All the experiments were carried out at 310 °K temperature. For each 1D spectrum, 4 dummy scans were used with receiver gain of 203. The set of all 1D ^13^C free induction decays were processed with 10 Hz line broadening with exponential function. All 1D carbon NMR data, acquired with either of the instrument, were processed for base line and phase correction, and external TSP peak calibration by using qumulti, multi_integ TOPSPIN commands. The chemical shift values are in ppm with respect to TSP as the internal reference. Peak assignments for glucose and lactate carbons were confirmed from previously reported data^31,32^.

### Quantifying glycolytic activity of *P. falciparum* infected human erythrocytes

From the NMR spectra obtained with *P. falciparum* infected human erythrocytes, the peaks corresponding to all carbons of glucose and lactate were identified (Supplementary Fig. S1). However, C6 of glucose and C3 of lactate peak data were used for analysis since the corresponding signals were more prominent and easier to quantify. Absolute quantification of glucose was carried out by extrapolating the integral values obtained for 1 mM to 5 mM standard glucose solutions (Supplementary Fig. S1d). In case of lactate however, relative quantification was obtained using the raw integral values. Data from individual experiments were plotted to obtain linear fits and the rate of glucose utilization or lactate production were derived from the slope of the linear fits. Glycolytic activity of normal (uninfected) RBCs was also measured in a similar manner. Glycolytic activity of the parasite was profiled during the entire intrerythrocytic developmental cycle, using both glucose and lactate measurements. Glucose utilization by the iRBCs was plotted as micromoles of glucose depleted per minute. This value was obtained from the slope of linear fits for data from each 6 h NMR experiment, and was plotted against the time corresponding to the midpoint of the respective 6 h sampling period. Glycolytic activity profiling by monitoring lactate production was carried out in a similar manner, except that the raw integral values were used to generate the linear fits for lactate production for each 6 h experiment.

### Measuring parasite glycolytic activity in the presence of antimalarial compounds

In these experiments, NMR measurement from antimalarial compound treated cells was obtained for a short duration of 2 hours, and analysis was done using lactate data only. The raw integral values were plotted as linear fits, and the ratio of the slope values derived from these fits for control and inhibitor treated parasites were used to assess the effect of antimalarial compounds on ring and trophozoite stage parasites. The parasitemia and progression of the parasite through the intrerythrocytic developmental cycle were monitored by microscopy using Giemsa stained culture smears.

## Electronic supplementary material

Supplementary Information

## References

[1] WHO World Malaria Report, http://www.who.int/malaria/publications/world-malaria-report-2017/en/ (2017).

[2] Miller LH, Ackerman HC, Su X-Z, Wellems TE (2013). Malaria biology and disease pathogenesis: insights for new treatments. Nat. Med..

[3] Salcedo-Sora JE, Caamano-Gutierrez E, Ward SA, Biagini GA (2014). The proliferating cell hypothesis: a metabolic framework for Plasmodium growth and development. Trends Parasitol..

[4] van Niekerk DD, Penkler GP, du Toit F, Snoep JL (2016). Targeting glycolysis in the malaria parasite *Plasmodium falciparum*. FEBS. J..

[5] van Schalkwyk DA, Priebe W, Saliba KJ (2008). The inhibitory effect of 2-halo derivatives of D-glucose on glycolysis and on the proliferation of the human malaria parasite *Plasmodium falciparum*. J. Pharmacol. Exp. Ther..

[6] Mehta M, Sonawat HM, Sharma S (2005). Malaria parasite-infected erythrocytes inhibit glucose utilization in uninfected red cells. FEBS. Lett..

[7] Klonis N (2013). Altered temporal response of malaria parasites determines differential sensitivity to artemisinin. Proc. Natl. Acad. of Sci. USA.

[8] Teuscher F (2010). Artemisinin induced dormancy in *Plasmodium falciparum*: Duration, recovery rates and implications in treatment failure. J. Infect. Dis..

[9] Teuscher F, Chen N, Kyle DE, Gatton ML, Cheng Q (2012). Phenotypic changes in artemisinin-resistant *Plasmodium falciparum* lines *in vitro*: evidence for decreased sensitivity to dormancy and growth inhibition. Antimicrob. Agents and Chemother..

[10] Bennett TN (2004). Novel, rapid, and inexpensive cell-based quantification of antimalarial drug efficacy. Antimicrob. Agents and Chemother..

[11] Baniecki ML, Wirth DF, Clardy J (2007). High-throughput *Plasmodium falciparum* growth assay for malaria drug discovery. Antimicrob. Agents and Chemother..

[12] Geary TG, Divo AA, Jensen JB (1983). An *in vitro* assay system for the identification of potential antimalarial drugs. J. Parasitol..

[13] Karl S, Wong RP, St Pierre TG, Davis TM (2009). A comparative study of a flow-cytometry-based assessment of *in vitro Plasmodium falciparum* drug sensitivity. Malar. J..

[14] Le Roch KG (2004). Global analysis of transcript and protein levels across the *Plasmodium falciparum* life cycle. Genome. Res..

[15] Olszewski KL, Llinás M (2011). Central carbon metabolism of *Plasmodium* parasites. Mol. Biochem. Parasitol..

[16] Slater AFG (1993). Chloroquine: Mechanism of drug action and resistance in *Plasmodium falciparum*. Pharmacol. Ther..

[17] Klayman D (1985). Qinghaosu (artemisinin): an antimalarial drug from China. Science.

[18] Chiodini PL (1995). Evaluation of atovaquone in the treatment of patients with uncomplicated *Plasmodium falciparum* malaria. J. Antimicrob. Chemother..

[19] Baragaña B (2015). A novel multiple-stage antimalarial agent that inhibits protein synthesis. Nature.

[20] Hoepfner D (2012). Selective and specific inhibition of the *Plasmodium falciparum* lysyl-tRNA synthetase by the fungal secondary metabolite cladosporin. Cell Host Microbe.

[21] Biamonte MA, Wanner J, Le Roch KG (2013). Recent advances in malaria drug discovery. Bioorg. Med. Chem. Lett..

[22] Haldar K, Bhattacharjee S, Safeukui I (2018). Drug resistance in *Plasmodium*. Nat. Rev. Microbiol..

[23] Srivastava IK, Morrisey JM, Darrouzet E, Daldal F, Vaidya AB (1999). Resistance mutations revel the atovaquone-binding domain of cytochrome *b* in malaria parasites. Mol. Microbiol..

[24] Talman AM, Domarle O, McKenzie FE, Frédéric A, Vincent R (2004). Gametocytogenesis: the puberty of *Plasmodium falciparum*. Malar. J..

[25] Blasco B, Leroy D, Fidock DA (2017). Antimalarial drug resistance: linking *Plasmodium falciparum* parasite biology to the clinic. Nat. med..

[26] Roth EJ (1990). *Plasmodium falciparum* carbohydrate metabolism: a connection between host cell and parasite. Blood Cells.

[27] Olszewski KL (2009). Host-parasite interactions revealed by *Plasmodium falciparum* metabolomics. Cell Host Microbe..

[28] Radfar A (2009). Synchronous culture of *Plasmodium falciparum* at high parasitemia levels. Nat. Protoc..

[29] Rivadeneira EM, Wasserman M, Espinal CT (1983). Separation and concentration of schizonts of *Plasmodium falciparum* by Percoll gradients. J. Protozool..

[30] Smilkstein, M., Sriwilaijaroen, N., Kelly, J. X., Wilairat, P., Riscoe, M. Simple and inexpensive fluorescence-based technique for high-throughput antimalarial drug screening. *Antimicrob. Agents Chemother*. **48**, 1803–1806 (2004). 10.1128/AAC.48.5.1803-1806.2004 PMC400546 15105138

[31] Lloyd, S. G., Zeng, H., Wang, P., Chatham, J. C. Lactate isotopomer analysis by 1H NMR spectroscopy: consideration of long-range nuclear spin-spin interactions. *Magn. Reson. Med*. **51**, 1279–1282 (2004). 10.1002/mrm.20075 15170850

[32] Pomin, V. H. Unravelling Glycobiology by NMR Spectroscopy., Glycosylation (Chapter. 04, Page 63–98). (ed Stefana Petrescu) (InTech, 2012).

