# Supplementary material for: Evaluating antimalarial efficacy by tracking glycolysis in Plasmodium falciparum using NMR spectroscopy
Source: Sci Rep. 2018 Dec 24;8:18076. doi: 10.1038/s41598-018-36197-3 (PMC6305384; doi:10.1038/s41598-018-36197-3)
Supplement: Supplementary file 1 — Supplementary Information [file 41598_2018_36197_MOESM1_ESM.docx]

**Title:** Evaluating antimalarial efficacy by tracking glycolysis in *Plasmodium falciparum* using NMR spectroscopy.

Rupali Shivapurkar^1^, Tejashri Hingamire^1,2^, Akshay S. Kulkarni^3^, P. R. Rajamohanan^4^, D. Srinivasa Reddy^2,3^, Dhanasekaran Shanmugam^1,2*^

**^1^** Biochemical Sciences Division, CSIR-National Chemical Laboratory, Pune, India

**^2^** Academy of Scientific and Innovative Research (AcSIR), New Delhi, India

**^3^** Organic Chemistry Division, CSIR-National Chemical Laboratory, Pune, India

**^4^** Central NMR facility, CSIR-National Chemical Laboratory, Pune, India

**^*^** Address correspondence to Dhanasekaran Shanmugam, [d.shanmugam@ncl.res.in](mailto:d.shanmugam@ncl.res.in)

***Supplementary Figure Legends***

**Supplementary Figure 1**. *U^13^C-glucose and U^13^C-lactate* *NMR profiles obtained from P. falciparum infected RBCs*. (**a**) Stack plot of the first (6.5 min; bottom panel) and last (325 min; top panel) ^13^C NMR spectrum obtained from a typical NMR measurement with live *P. falciparum* infected RBCs on the Bruker Avance 500MHz spectrometer. The asterisks in the top panel indicate the peaks corresponding to the three lactate carbons. For experimental details see Materials and Methods section in main text. (**b**) NMR peak profiles for the various carbons of lactate (a to c) and glucose (d to k) is shown. a, C1-Lactate; b, C2-Lactate; c, C3-Lactate; d, β-C1-D-Glucose; e, α-C1-D-Glucose; f, β-C3-D-Glucose; g, β-C2-D-Glucose; h, α-C3-D-Glucose; i, α-C2-D-Glucose, α,β-C4-D-Glucose; j, α,β-C5-D-Glucose; k, α,β-C6-D-Glucose. (**c**), Structure of glucose and lactate annotated with the various carbons highlighted in **b**. (**d**) Standard graph showing the linear fit of NMR measurements for different concentrations of glucose. The linear fit equation was used to derive absolute quantification of the rate of glucose utilized by iRBCs. Representative data from one independent experiment is shown.

**Supplementary Figure 2**. *Evaluating the* *viability of parasites recovered after NMR measurements*. In order to ascertain that the ring and trophozoite stage parasites have remained viable during the NMR experiments, following NMR acquisition, the cultures were recovered from the NMR tubes aseptically and then cultured under optimal conditions for up to 72 hps (when the parasites will be in their 2^nd^ intraerythrocytic cycle). The parasites were observed by microscopy for normal morphology and development at various time points. The top panel shows the control cultures, which were not subject to NMR experiment. The middle and bottom panel shows the profile of ring and trophozoite stage parasites, which were subject to 2 h NMR experiments respectively. The presence of well developed trophozoites at the 72 h time point with comparable Parasitemia (~15%) to control cultures indicates that the parasites were not compromised in their viability during the course of the NMR experiments.

**Supplementary Figure 3**. *Potency of the various antimalarial compounds used in this study*. The *EC*_50_ values for chloroquine, atovaquone, cladosporin, DDD107498 (a 2,6-disubstituted quinoline-4-carboxamide scaffold^19^) and artemisinin were calculated from the fits derived from % growth inhibition *vs* antimalarial compound concentration plots. A previously published protocol^30^ was followed for the assay used to determine the *EC*_50_ values. The raw data was processed using Microsoft Excel spreadsheets for calculating dose response and *EC*_50_ estimation. The number of replicates used in all assays n=2. The estimated *EC*_50_ values are similar to that reported previously for these molecules and are shown above individual plots.

**Supplementary Figure 4**. *Plots depicting the raw NMR peak integral values as measured from the parasites treated with various inhibitors*. The integral value of lactate C3 is plotted *vs* time for ring and trophozoite stage parasites, with and without treatment of the different antimalarial compounds. Data from replicate experiments is shown as follows; **a** and **b**, 1 µM chloroquine, **c** and **d**, 0.1 µM chloroquine, **e** and **f**, 1 µM atovaquone, **g** and **h**, 1 µM cladosporin, **i** and **j**, 1 µM DDD107498, **k** and **l**, 1 µM artemisinin (5 h treatment before NMR measurement) and **m** and **n**, 1 µM artemisinin (10 h treatment before NMR measurement). **a** and **b** data is overlaid and shown in Fig. 3**b**. The fold change in rate of lactate production between antimalarial compound treated and untreated samples from the various experiments is shown in main Fig. 3**c** and Fig. 4**a** to **f**, respectively.

**Supplementary Figure 5**. *Morphological changes in P. falciparum associated with drug treatment*. (**a**) The normal morphology of ring and trophozoite stage parasites from control cultures is shown in the microscopic images corresponding to the various time periods during which the NMR measurements were taken. Ring (top panel) and trophozoite (bottom panel) stage parasites are shown. (**b**), Morphology of antimalarial compound treated ring and trophozoite stage parasites at the start (5 and 29 hps) and end (7 and 31) of NMR measurements, respectively. From the images shown in panel **a** and **b**, it is clear that, unlike in case of trophozoites, the morphology of the ring stage parasite is not significantly altered by antimalarial compound treatment during the experimental time period.

**Supplementary Figure 6**. *Monitoring the* *death of P. falciparum parasites treated with chloroquine.* Giemsa stained smears showing the morphology of control and chloroquine (0.1 µM and 1 µM) treated ring and trophozoite stage parasites after 24 h and 48 h of continuous drug treatment. Chloroquine treatment was started immediately after sorbitol synchronization, which is used to enrich the culture for ring stage parasites. This experiment shows that eventhough the viability of ring stage parasites is not significantly affected by chloroquine, their maturation into trophozoite and continuation of the intraerythrocytic developmental cycle is severely inhibited.

**Supplementary Figure 1***
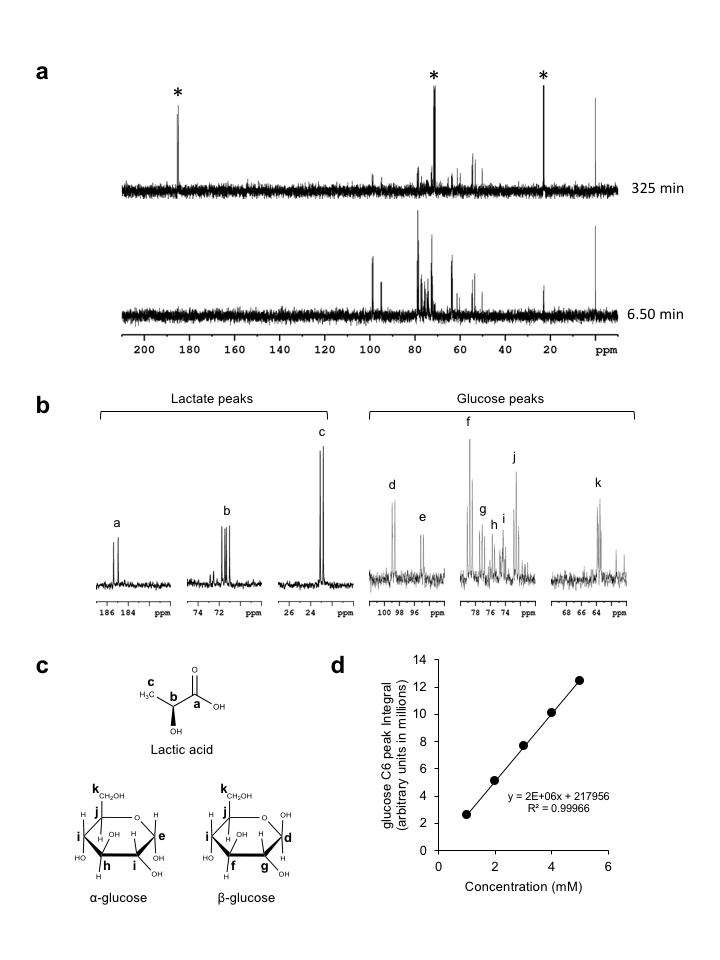
*

**Supplementary Figure 2**

*
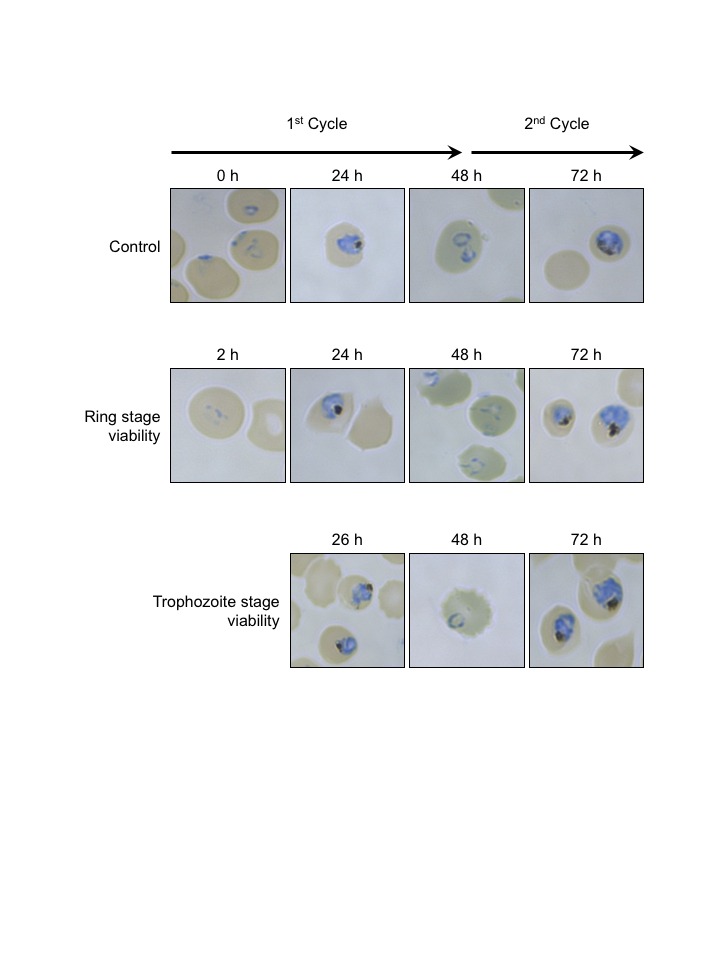
*

**Supplementary Figure 3**

*
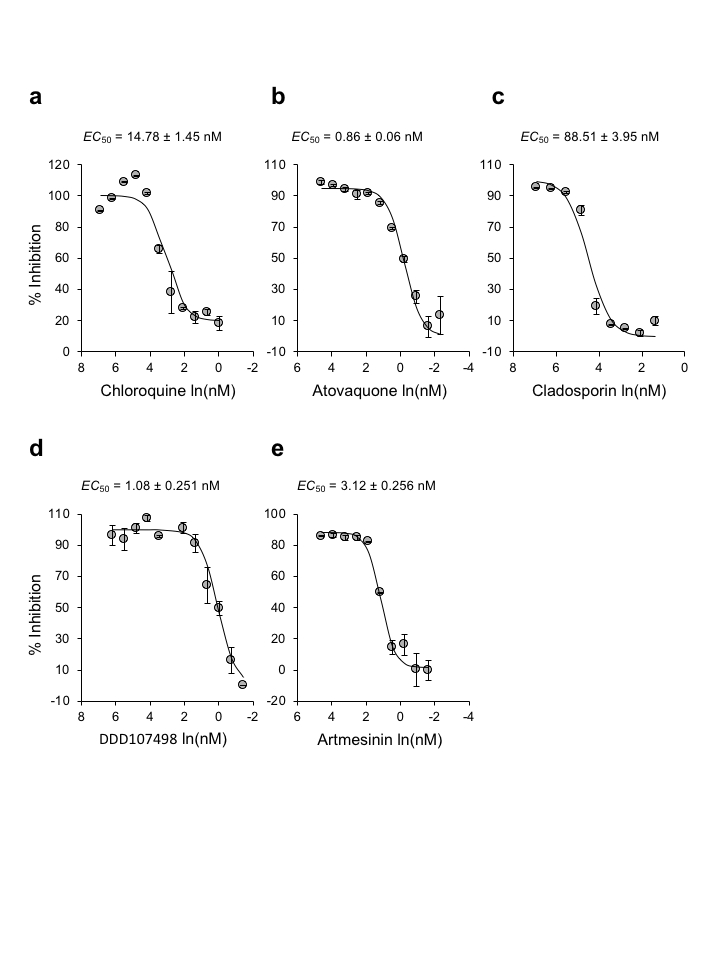
*

**Supplementary Figure 4**

*
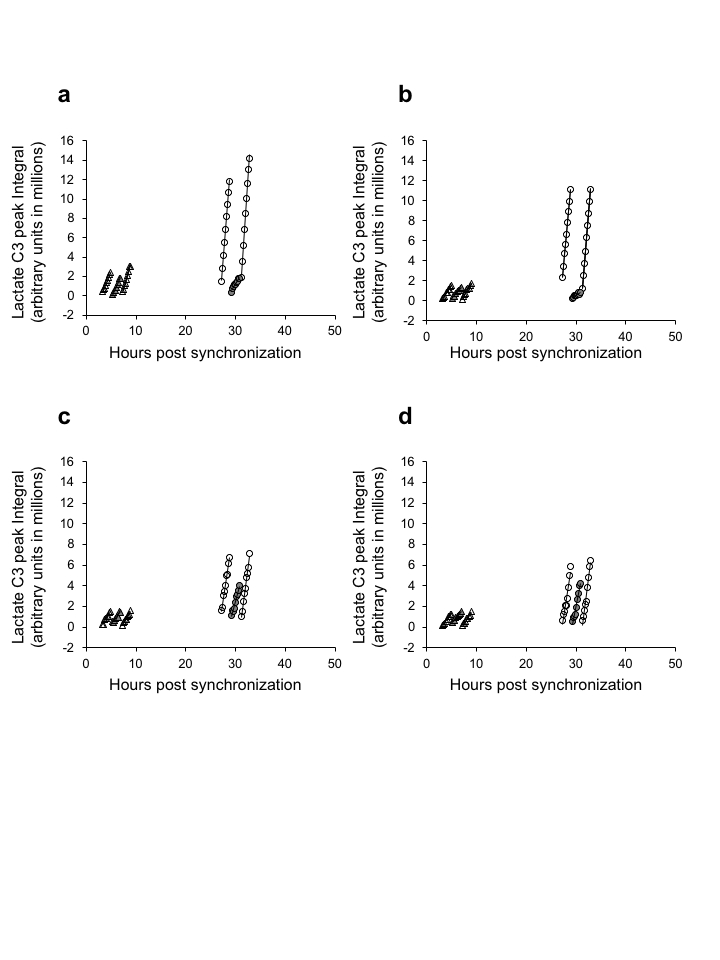
*

**Supplementary Figure 4 continued**

*
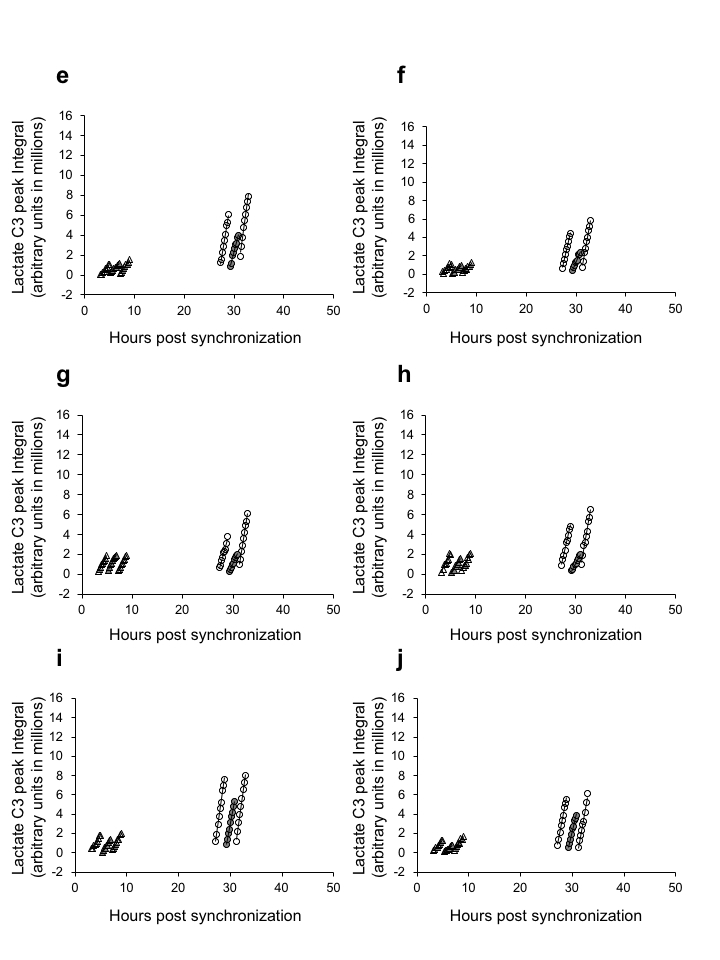
*

**Supplementary Figure 4 continued**

*
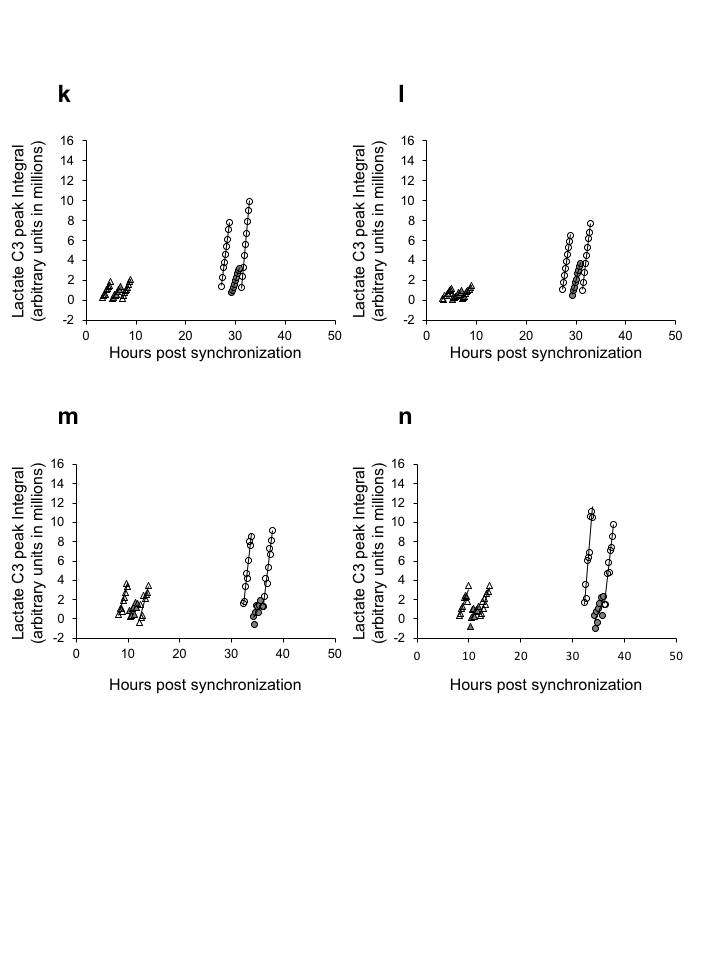
*

**Supplementary Figure 5**

*
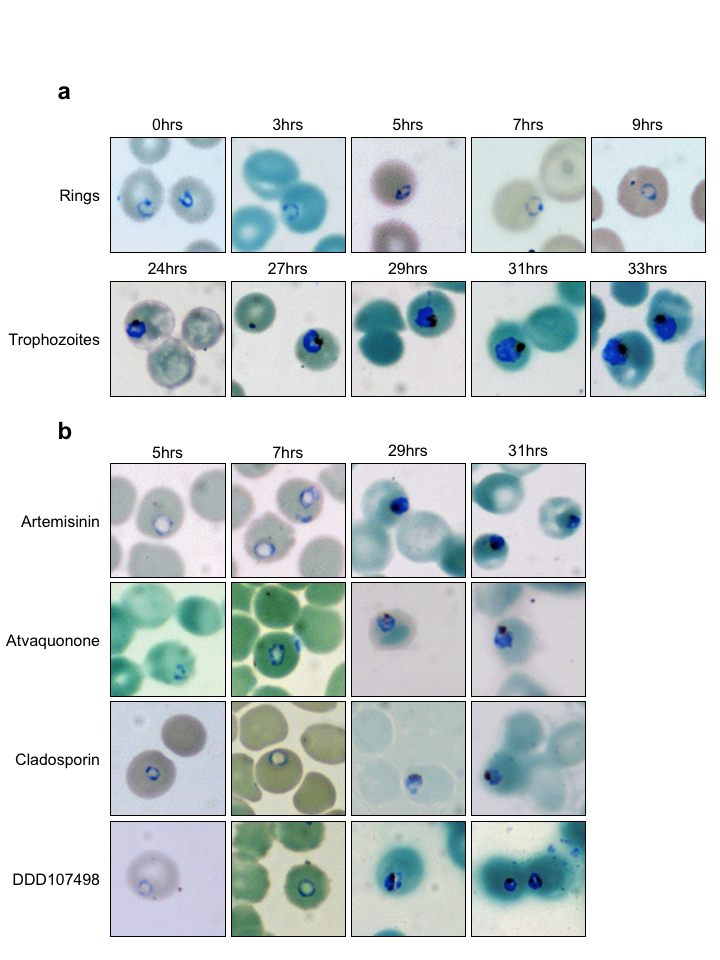
*

**Supplementary Figure 6**

*
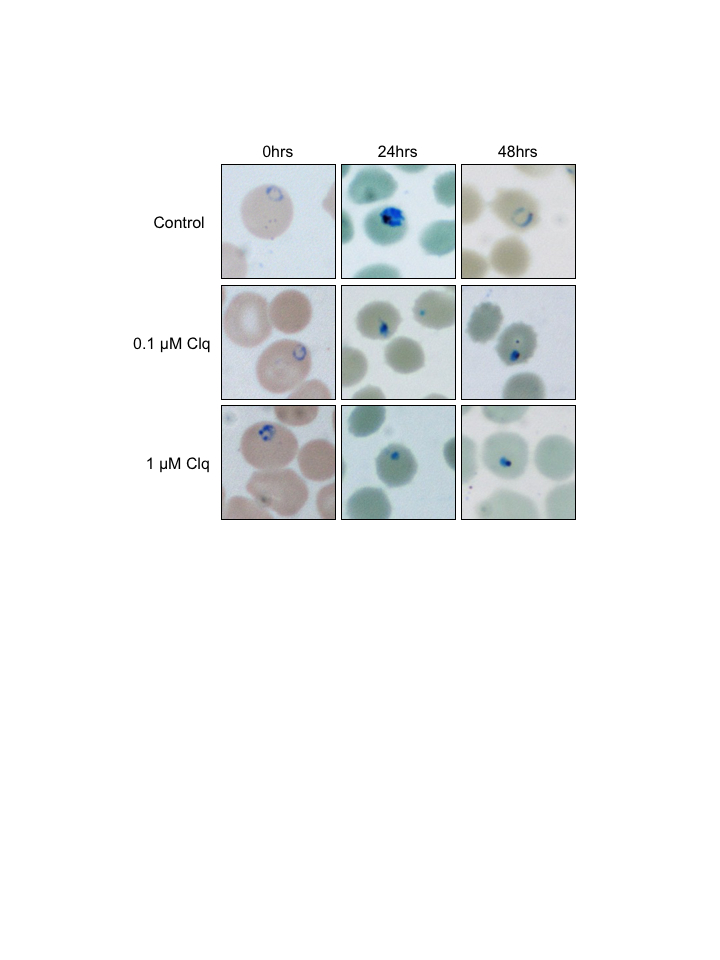
*
